# In Vitro Evaluation of the Protective Efficacy of *Crocus sativus* L. Waste for the Sustainable Development of Bioactive Phytocomplexes

**DOI:** 10.3390/molecules30142894

**Published:** 2025-07-08

**Authors:** Alessia Galante, Francesca Corsi, Emily Cioni, Mauro Di Stasi, Maria Anna Maggi, Silvia Bisti, Ilaria Piano, Claudia Gargini

**Affiliations:** 1 Department of Pharmacy, University of Pisa, Via Bonanno 6, 56126 Pisa, Italy; alessia.galante@phd.unipi.it (A.G.); francesca.corsi@farm.unipi.it (F.C.); emily.cioni@phd.unipi.it (E.C.); mauro.distasi@farm.unipi.it (M.D.S.); 2 National Institute of Biostructures and Biosystems (INBB), Via Medaglie d’Oro 305, 00136 Rome, Italy; s.bisti@team.it; 3 Hortus Novus Srl, Via Campo Sportivo 2, 67050 Canistro, Italy; m.maggi@hortusnovus.it; 4 Department of Physical and Chemical Sciences, University of L’Aquila, 67100 Coppito, Italy; 5 CISUP, Center for Instrument Sharing of the University of Pisa, University of Pisa, Lungarno Pacinotti 43/44, 56126 Pisa, Italy; 6 Interdepartmental Center for Nutraceutical Research and Foods for Health (NUTRAFOOD), University of Pisa, 56126 Pisa, Italy

**Keywords:** *Crocus sativus*, ARPE-19, saffron waste, tepal extract, phenols

## Abstract

Saffron, branded as Repron^®^, is effective in slowing the progression of several neurodegenerative diseases. Its production, however, requires specific cultivation techniques and procedures that, together with low yields, make it expensive. To address this challenge, hydroponic cultivation has been adopted. Previous studies have shown that hydroponically cultivated saffron and Repron^®^ share comparable chemical compositions and neuroprotective effects under oxidative stress conditions. In this study, we evaluated the protective properties of extracts derived from *Crocus sativus* L. waste, compared with those of saffron derived from stigmas. Human retinal pigment epithelium (ARPE-19) cells were pre-treated with extracts of various plant waste fractions before being subjected to three stress conditions: H_2_O_2_-induced oxidative stress (500 μM, 3 h), lipopolysaccharide (LPS; 0.25 mg/mL, 24 h), and hyperglycemia (25 mM glucose, 96 h). Saffron Repron^®^ served as a positive control. The results revealed that the extract derived from *C. sativus* waste had superior protective effects against oxidative stress and inflammation by preserving the state of the mitochondria and tight junctions (ZO-1); conversely, the tepal extract alone was more effective under hyperglycemic conditions by also modulating acrolein levels. These results suggest that different plant fractions contain bioactive compounds with specific protective actions, which together lead to increased cell survival.

## 1. Introduction

Saffron—a spice known for centuries for its beneficial and culinary properties—is obtained by drying the stigmas of *Crocus sativus* L. (family Iridaceae). It is a perennial bulb found mainly in Asia and European countries bordering the Mediterranean, particularly Greece, Spain, and Italy [1]**,** where the most valuable species are grown in Abruzzo and Sardinia [2]. The quality of saffron is determined by evaluating its taste, aroma, and color—characteristics that are influenced by several factors such as soil, climate, rainfall, harvest time, and post-harvest treatments. Sensory evaluations are complemented by the nutraceutical properties of the spice. Among the most represented molecules, picrocrocin (the precursor glycoside of safranal) contributes to bitterness and is responsible for its flavor, while safranal (a monoterpene aldehyde formed by the hydrolysis of picrocrocin during drying and storage [3]) imparts the characteristic aroma of saffron and is involved in several pharmacological activities [4]. Furthermore, crocins—the glycosylated esters of crocetin with glucose, gentiobiose, neapolitanose, or triglucose—are water-soluble carotenoids that are responsible for its yellowish color. Quantifying these compounds is important in establishing the commercial quality of saffron and, consequently, its final price [5]; recently, it has been found that the ratio of crocins provides saffron with distinctive neuroprotective properties [6]**.** This has led to the development of a patent for Repron^®^ saffron (patent: “Compositions based on saffron for the prevention and/or treatment of degenerative eye disorders”, 2015 (W02015/145316)) [7]. In vivo and in vitro models have highlighted the ability of Repron^®^ saffron to prevent and slow down the progression of neurodegenerative diseases associated with oxidative stress and inflammation, including at the retinal level [8]. Furthermore, crocins and safranal have inhibitory effects on amyloid-β (Aβ) plaque aggregation, tau protein hyperphosphorylation (conditions that occur in diseases such as Alzheimer’s and Parkinson’s), and NF-kB pathway activation by reducing oxidative stress and exerting anti-inflammatory action [9,10]. Similarly, crocetins act by inhibiting NF-kB activation and p53 expression in the hippocampus, decreasing pro-inflammatory cytokine secretion and increasing the production of anti-inflammatory cytokines [11]. The effectiveness of saffron treatment has also been demonstrated in degenerative retinal diseases: studies of in vivo models and patients with age macular degeneration (AMD) have shown that saffron can protect retinal photoreceptors from stress while preserving their structure and function [12]. Other studies have demonstrated the efficacy of safranal in attenuating retinal degeneration in animal models of retinitis pigmentosa (RP): the results showed that administering safranal preserved both the number and function of photoreceptors, highlighting (following electroretinography) a better response under both photopic and scotopic conditions [13]. Given the great potential of saffron as a nutraceutical product, questions have begun to be raised about the high cost of its production, which is characterized by low yields. As a result, saffron-based therapies would have significant production costs, making them unaffordable for most of the population. For saffron production, the stigmas are separated from the rest of the flower, and the remaining parts (tepals, stamens, and style) represent an agricultural bioresidue: to produce 1 kg of saffron, about 63 kg of bioresidue is generated [14]. The flowers, especially the tepals, are the main by-product of saffron processing; they are produced in high quantities and discarded as waste. However, saffron tepals also contain various compounds, such as mineral agents, anthocyanins, flavonoids, glycosides, and alkaloids. Therefore, the waste products of saffron production could be useful resources for various purposes [15]. Several studies have described various pharmacological properties of saffron tepals, such as antibacterial [16], antispasmodic, antihypertensive [17], immunomodulatory [18], antitussive [19,20], antidepressant [21], and antinociceptive [22] activities. Given these properties, saffron tepals can be used as an alternative or integrative medicine in some diseases; for example, in traditional Asian medicine, saffron tepal extracts are consumed as an antispasmodic, stomachic, anxiety curative, anticancer, and antidepressant agent.

This study aimed to evaluate the potential protective effect of both saffron tepals and waste extracts in in vitro models of retinal epithelium using human ARPE-19 cells. Damage (either environmental or genetic) to the retinal pigment epithelium (RPE) can trigger or be a cause of retinal neurodegenerative diseases, such as diabetic retinopathy (DR) [23], AMD [24], and RP [25]. Here, we show that extracts obtained from different saffron production wastes appear to protect ARPE-19 from damage in a bioactive molecule content-dependent and stress-applied manner.

## 2. Results

### 2.1. Chemical Composition of Saffron Extracts

**Figure 1:**
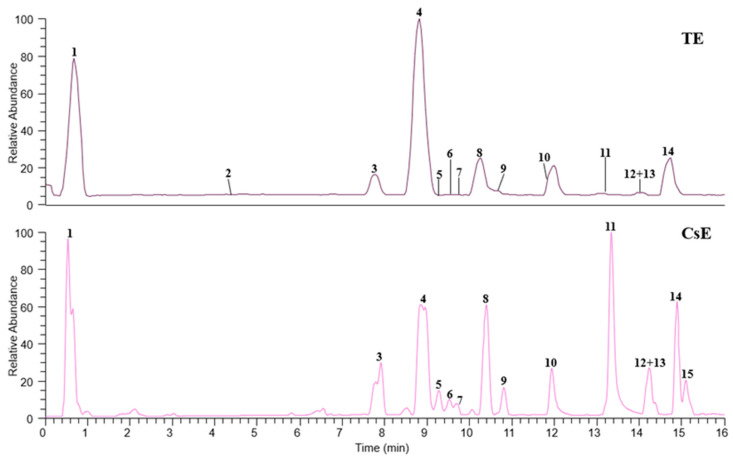
HR-LC-MS profiles of saffron tepal extract (TE) and saffron waste extract (CsE), recorded in negative ESI mode. Each number corresponds to an annotated molecule listed in Table 1.

Saffron waste extract (CsE) and saffron tepal extract (TE) showed chromatographic profiles rich in flavonoid glycosides (Figure 1), confirmed by HR-MS^2^ data and a mass error of <5 ppm on the experimental molecular mass, as reported in Table 1. In particular, kaempferol, isorhamnetin, and quercetin were identified in the form of aglycones (**10**–**12**) or bound with one to three monosaccharides (**2**–**9**) as major components, in accordance with previous studies [2,14,26,27]. Based on the loss of saccharide residues, these compounds were identified as kaempferol tri-*O*-glucoside (**2**), kaempferol *O*-sophoroside (**4**), kaempferol *O*-rutinoside (**6**), and kaempferol *O*-glucoside (**8**) [28]. Quercetin *O*-sophoroside (**3**) and quercetin *O*-glucoside (**5**) were annotated by the presence of a base ion peak at *m*/*z* 300.03, corresponding to the aglycone portion of quercetin (**10**). Compounds **7** and **9** were identified as isorhamnetin (**12**) derivatives, exhibiting both the product ion at *m*/*z* 315.05 and the loss of a rutinose unit (−308 u) or a glucoside portion (−162 u), respectively, leading to the identification of **7** as isorhamnetin *O*-rutinoside and **9** as isorhamnetin *O*-glucoside [29]. An organic acid (**1**) and three hydroxylated fatty acids (**13**–**15**) were also detected. Compound **1** was annotated as citric acid due to the deprotonated ion [M − H]^−^ at *m*/*z* 191.0192 and the product ion [M − H − 18]^−^ at *m*/*z* 173.01, corresponding to the loss of a water molecule [30]. Compounds **13**, **14**, and **15** showed the consecutive neutral loss of two molecules of water and were identified as trihydroxyoctadecadienoic acid and trihydroxyoctadecenoic acid isomers [31,32].

**Figure 2:**
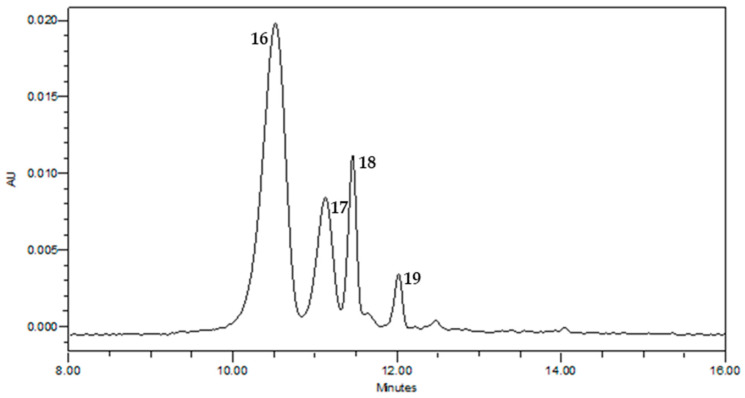
HPLC-DAD chromatogram at 530 nm of saffron tepal extract (TE). Each number corresponds to an annotated molecule listed in Table 2.

Below (Figure 2), an HPLC-DAD chromatogram of the tepals extract is shown relative to anthocyanins, whose signals are more intense because the sample amount is larger than that analyzed using LC-MS. The compounds were identified using the same method used for the analytes in Table 1. Four anthocyanins (**16**–**19**, Figure 2)—previously reported in the literature as saffron bioresidue constituents by Serrano-Díaz et al., 2014 [14] and Cusano et al., 2017 [2]—were identified and confirmed using HR-MS^2^ data. Compounds **16** and **18** were annotated as delphinidin 3,5-di-*O*-glucoside (**16**) and delphinidin 3-*O*-glucoside (**18**), as deduced by a base ion peak at *m/z* 303.05 due to the aglycon portion of delphinidin and generated by the loss of one or two glucose residues, respectively (Table 2). Similarly, compounds **17** and **19** were annotated as petunidin 3,5-di-*O*-glucoside (**17**) and petunidin 3-*O*-glucoside (**19**), showing a base ion peak at *m*/*z* 317.06 corresponding to the aglycon portion of petunidin [27].

### 2.2. In Vitro Results

To evaluate the protective efficacy of extracts obtained from saffron, three different models of cellular damage were used, oxidative stress, inflammation, and hyperglycemia, following the protocols shown in the Materials and Methods section (see below. These stress models were chosen to identify a correlation between the pathways activated by cells in response to damage and the bioactive molecules present in the various extracts.

#### 2.2.1. Different Saffron and Waste Extracts Protect Against Oxidative Stress

**Figure 3:**
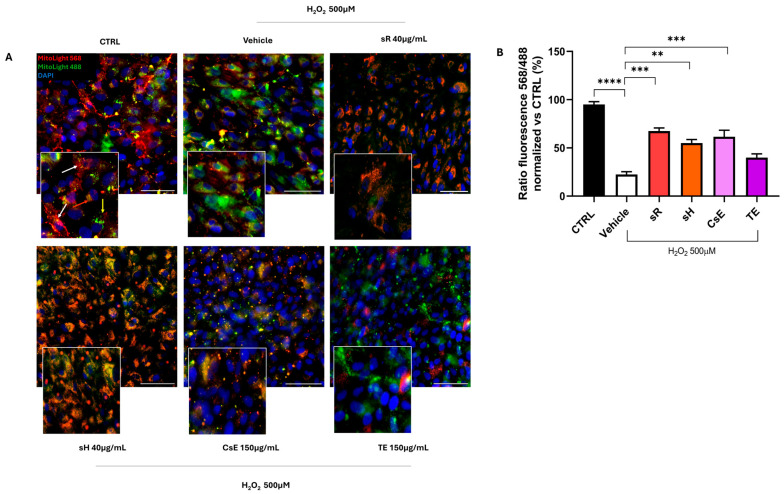
Extract efficacy assays following oxidative stress induced by H_2_O_2_ damage. Panel (**A**) shows cells stained with MitoLight, a mitochondrial marker that shifts from red to green in response to changes in membrane potential. In CTRL conditions, untreated cells exhibit intact mitochondrial membranes, as indicated by the red fluorescence, meaning that the dye remains localized within the mitochondria (the arrows indicate the mitochondria labelled in different colours according to their oxidative state). The vehicle, representing the pathological control, consists of cells exposed to H_2_O_2_ 500 µM in culture medium. These cells display significant mitochondrial depolarization, as evidenced by the shift from red to green/yellow fluorescence. Panel (**B**) quantifies the MitoLight fluorescence ratio (585/488 nm) using ImageJ Java 8, normalized to the control condition. The vehicle confirms the detrimental effect of oxidative stress and shows a significant reduction in mitochondrial membrane integrity. However, treatment with the saffron extracts partially restores mitochondrial potential, as indicated by the statistical significance of the results (** *p* < 0.01; *** *p* < 0.001; **** *p* < 0.0001. Scale bars = 25 µm.

In Figure 3, the results obtained for ARPE-19 cells after damage induced by incubation in a medium containing 500 μM of H_2_O_2_ are shown. Panel A displays an assessment of the mitochondrial damage, and it is possible to observe how the healthy control cells (CTRL) exhibit predominantly red staining, indicating proper mitochondrial membrane integrity. In contrast, damaged cells treated with vehicle alone (vehicle) show predominantly green staining, indicative of mitochondrial damage and dysfunction. In the panels showing the various treatments, the two stains vary depending on the origin of the extract. Cells with more prominent red staining are those treated with saffron Repron^®^ (sR), while cells treated with hydroponic saffron (sH) show a certain degree of overlap between the two stains, with a shift from red to yellow. Meanwhile, cells treated with whole-flower extract (CsE) and tepal extract (TE) exhibit predominantly green staining. These observations, summarized in Panel B, indicate that in the case of oxidative damage, the saffron Repron^®^ extract significantly preserves mitochondrial integrity and functionality, followed by hydroponic saffron, also classified as “Repron^®^.”

**Figure 4:**
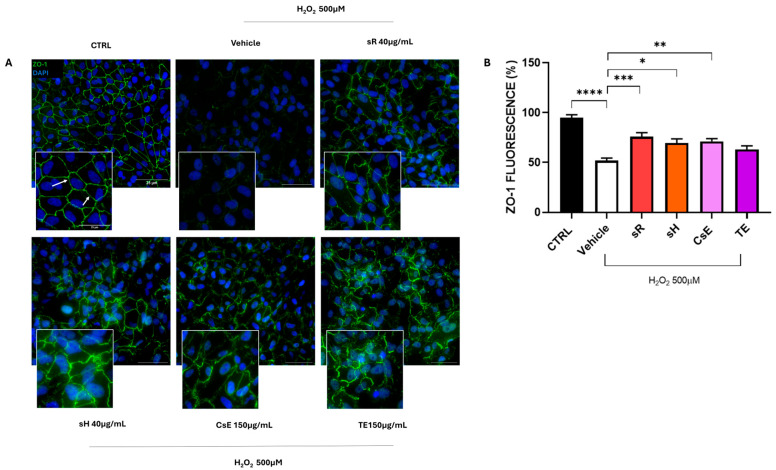
Tight junction integrity of different extracts after H_2_O_2_ damage. (**A**) shows ZO-1 (zonula occludens-1) immunofluorescence (green, indicated by white arrows), a key marker of tight junctions fundamental for RPE (retinal pigment epithelium) barrier integrity. The CTRL condition (untreated cells) displays continuous ZO-1 staining outlining cell borders, reflecting well-preserved tight junctions. In contrast, the vehicle group (cells exposed to H_2_O_2_ 500 µM) exhibits disrupted ZO-1 staining, indicating a loss of tight junction integrity. Treatment with different saffron-based extracts—Repron^®^ saffron extract (sR, 40 µg/mL), hydroponic saffron extract (sH, 40 µg/mL), whole-saffron-flower extract (CsE, 150 µg/mL), and saffron tepal extract (TE, 150 µg/mL)—partially restores the ZO-1 signal at the cell boundaries, suggesting a protective effect against oxidative stress. Cell nuclei are counterstained with DAPI (blue). Scale bar: 25 µm. (**B**) presents the quantification of ZO-1 fluorescence (expressed as a percentage of the CTRL), analyzed via one-way ANOVA (mean ± SEM, n = 4). The vehicle confirms the detrimental impact of oxidative stress, showing a marked reduction in ZO-1 expression. However, cells treated with saffron extracts exhibit significantly improved tight junction preservation (* *p* < 0.05; ** *p* < 0.01; *** *p* < 0.001; **** *p* < 0.0001).

**Figure 5:**
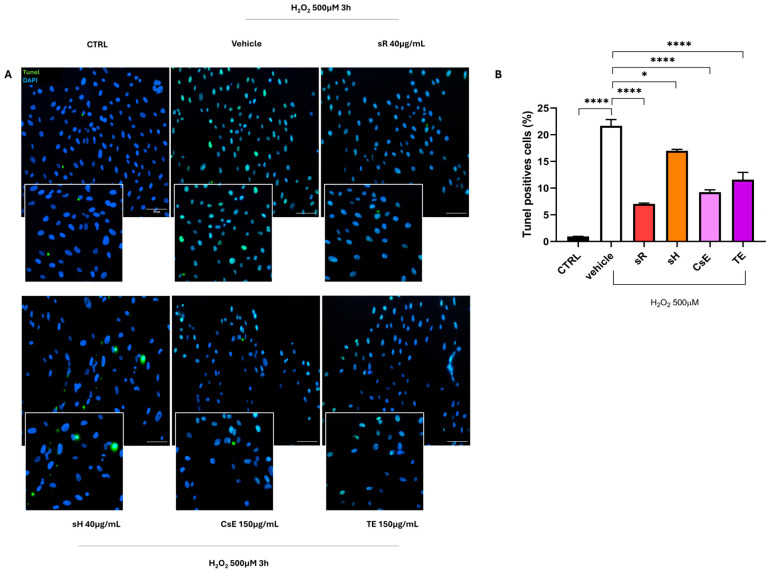
Anti-apoptotic efficacy of saffron extracts following oxidative stress induced by H_2_O_2_ damage. Panel (**A**) shows representative images of TUNEL staining (green), which detects the DNA fragmentation characteristic of apoptotic cells, following 500 µM H_2_O_2_ exposure for 3 h. The CTRL group (untreated cells) exhibits minimal TUNEL staining, indicating low apoptosis levels. By contrast, the vehicle group (exposed to H_2_O_2_ alone) displays a marked increase in TUNEL-positive nuclei. Treatment with saffron-based extracts Repron^®^ (sR, 40 µg/mL), hydroponic saffron extract (sH, 40 µg/mL), whole-saffron-flower extract (CsE, 150 µg/mL), and saffron tepal extract (TE, 150 µg/mL) reduces TUNEL staining, suggesting a protective effect against oxidative damage. Cell nuclei are counterstained with DAPI (blue). Scale bar = 100 µm. Panel (**B**) shows the quantification of TUNEL-positive cells (percentage), an indicator of apoptotic extent. The data are presented as mean ± SEM (n = 4). One-way ANOVA was performed to assess statistical significance. The vehicle confirms the harmful impact of oxidative stress, showing a substantial increase in apoptosis. However, cells treated with saffron extracts exhibit significantly reduced TUNEL positivity (* *p* < 0.05; **** *p* < 0.0001).

The different capacities of the various extracts to protect ARPE-19 cells from oxidative damage also emerge in Figure 4, which illustrates the integrity of cell junctions through the labeling of the tight junction-specific protein zonula occludens-1 (ZO-1). As shown in Panel A, the control group (CTRL) cells exhibit a well-defined green label along the cell membrane, highlighting the typical intersections of epithelial cells. Following exposure to oxidative stress, this label almost completely disappears in cells treated only with the vehicle, indicating a loss of junction integrity. In contrast, the panels related to treatments with different extracts show partial or complete preservation of the green ZO-1 label, suggesting a certain degree of protection against stress. The protective effect of the extracts was further quantified by measuring the fluorescence intensity of ZO-1. The bar graph in Figure 4B shows a significant protective effect for the sR, sH, and CsE extracts, while no statistically significant levels were reached for the extracts obtained from tepals alone (TE). The protective effect of saffron extracts on mitochondrial status, as well as on the preservation of cell junctions, reduces the apoptotic process by which cells are destined to die. Figure 5 shows the results obtained through TUNEL analysis, where Panel A displays a reduction in green labeling, indicating TUNEL+ cell nuclei, and Panel B quantifies the number of TUNEL+ cells expressed as a percentage. The bar graph in Panel B shows that treatment with the different tested extracts significantly reduces apoptotic cells.

#### 2.2.2. Different Saffron and Waste Extracts Protect from Inflammation

**Figure 6:**
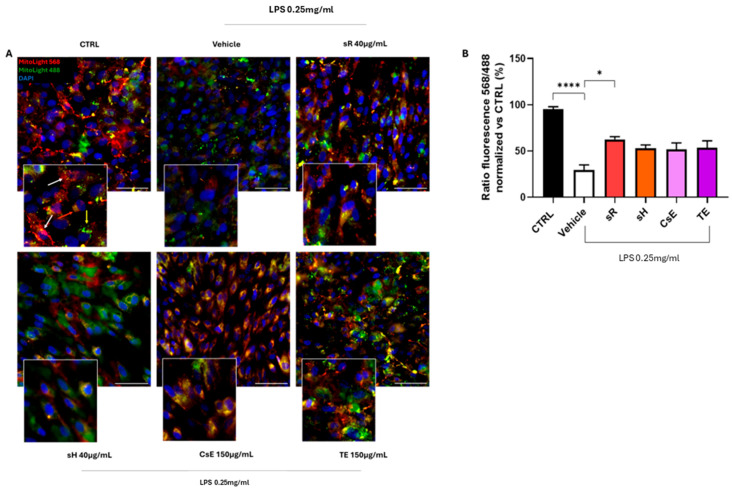
Activity of mitochondrial integrity of different extracts after LPS damage. Panel (**A**) shows representative images of cells stained with MitoLight. The CTRL condition (untreated cells) displays predominantly red fluorescence, indicative of intact mitochondrial membranes (the arrows indicate the mitochondria labelled in different colours according to their oxidative state). Exposure to LPS (0.25 mg/mL) leads to partial mitochondrial depolarization, visible as a shift toward green/yellow fluorescence. By contrast, treatment with saffron-based extracts—Repron^®^ saffron extract (sR, 40 µg/mL), hydroponic saffron extract (sH, 40 µg/mL), whole-saffron-flower extract (CsE, 150 µg/mL), and saffron tepal extract (TE, 150 µg/mL)—helps preserve mitochondrial membrane integrity. Cell nuclei are counterstained with DAPI (blue). Scale bar = 25 µm. Panel (**B**) presents the MitoLight fluorescence ratio (568/488 nm) normalized to the CTRL condition, analyzed using ImageJ. Data are expressed as mean ± SEM (n = 4). Statistical significance was determined via one-way ANOVA (* *p* < 0.05, **** *p* < 0.0001).

**Figure 7:**
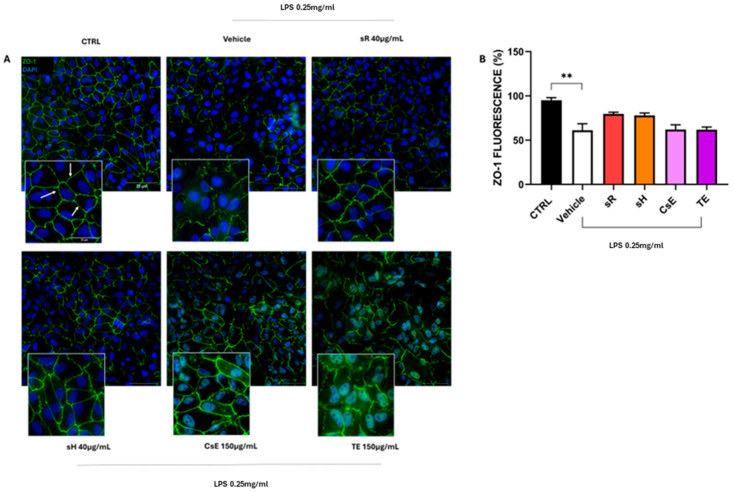
Activity of tight junction integrity of different extracts after LPS damage. (**A**) shows immunostaining for ZO-1 (green, indicated by white arrows). The CTRL (untreated) condition exhibits continuous ZO-1 staining outlining cell borders. By contrast, the vehicle group (exposed to LPS 0.25 mg/mL) demonstrates disrupted ZO-1 distribution, indicative of compromised tight junctions. Treatment with saffron-based extracts—Repron^®^ saffron extract (sR, 40 µg/mL), hydroponic saffron extract (sH, 40 µg/mL), whole-saffron-flower extract (CsE, 150 µg/mL), and saffron tepal extract (TE, 150 µg/mL)—partially restores ZO-1 localization at cell boundaries, suggesting a protective effect against inflammatory stress. Cell nuclei are counterstained with DAPI (blue). Scale bar = 25 µm. (**B**) quantifies ZO-1 fluorescence (percent of CTRL), serving as a measure of tight junction integrity. Data are expressed as mean ± SEM (n=4) and analyzed via one-way ANOVA. (** *p* < 0.01).

**Figure 8:**
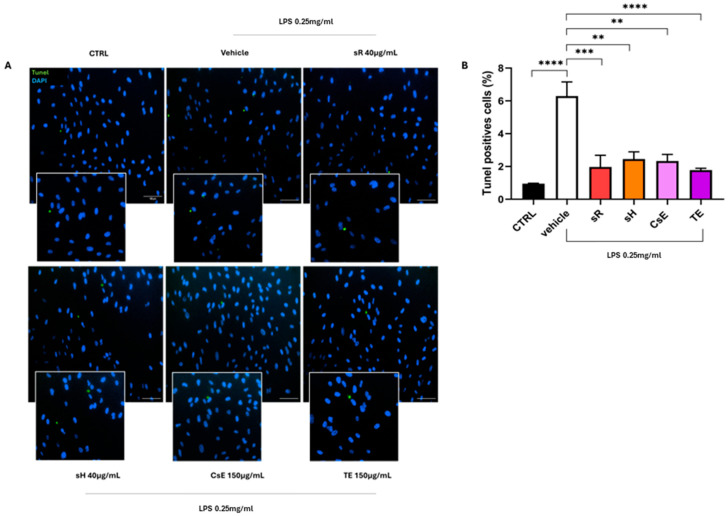
Saffron extract efficacy under LPS-induced inflammation: TUNEL assay. (**A**) shows representative images of TUNEL staining (green) after LPS (0.25 mg/mL) exposure for 24 h. The CTRL (untreated) group displays minimal TUNEL positivity, whereas the vehicle (LPS alone) group shows an increase in apoptotic cells. Treatment with saffron-based extracts—Repron^®^ saffron extract (sR, 40 µg/mL), hydroponic saffron extract (sH, 40 µg/mL), whole-saffron-flower extract (CsE, 150 µg/mL), and saffron tepal extract (TE, 150 µg/mL)—reduces TUNEL staining, suggesting a protective effect against inflammatory stress. Cell nuclei are counterstained with DAPI (blue). Scale bar = 100 µm. (**B**) quantifies the TUNEL-positive cells (percent), reflecting the degree of apoptosis. Data are expressed as mean ± SEM (n=4) and analyzed via one-way ANOVA (** *p* < 0.01; *** *p* < 0.001; **** *p* < 0.0001).

Figure 6 shows the results obtained for ARPE-19 cells following damage induced by incubation in a medium containing 0.25 mg/mL of LPS. In this case, Panel A illustrates the evaluation of the effectiveness of extracts obtained from saffron and its by-products in protecting cells from mitochondrial damage induced by LPS treatment. The healthy control (CTRL) cells exhibit predominantly red labeling, indicating mitochondrial membrane integrity. In contrast, damaged cells treated only with vehicles show predominantly green labeling, which indicates mitochondrial damage and dysfunction. These findings are fully consistent with the data shown in Figure 3. The labeling pattern changes on the extract source in the panels displaying the different treatments. Cells treated with saffron Repron^®^ (sR) exhibit more red labeling, whereas cells treated with hydroponic saffron (sH), whole-flower extract (CsE), and tepal-only extract (TE) predominantly show green labeling. These observations, summarized in Panel B, indicate that in the case of LPS-induced inflammation, the only extract with a significant ability to preserve mitochondrial integrity and functionality is saffron Repron^®^. Figure 7 illustrates the integrity of cell junctions through ZO-1 labeling. As shown in Panel A, CTRL cells display a green labeling pattern typical of epithelial morphology. Upon exposure to inflammatory stress (vehicle), ZO-1 labeling becomes highly fragmented and discontinuous. Although the panels for the various treatments show that labeling is partially preserved, indicating some degree of stress protection, no significant values are reached when ZO-1 fluorescence is quantified (Figure 7B). The protective effect of saffron extracts on mitochondrial status and the preservation of cell junctions, although not reaching statistical significance, still reduces the apoptotic process, as evidenced by the results obtained through TUNEL analysis. In Figure 8A, a reduction in green labeling can be observed, indicating TUNEL+ cell nuclei. In the bar graph in Panel B, it is clear that treatment with the different tested extracts protects the cells from apoptosis, significantly reducing their percentage.

#### 2.2.3. Different Saffron and Waste Extracts Protect from Hyperglycemia

**Figure 9:**
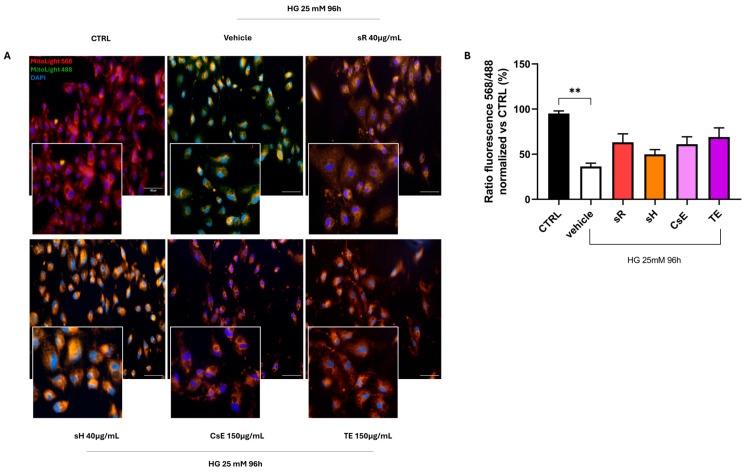
Saffron extract efficacy under hyperglycemic conditions in preserving mitochondrial membrane integrity. Panel (**A**) shows representative images of cells stained with MitoLight after glucose 25 mM exposure for 96 h. The CTRL group (normal glucose) displays predominantly red fluorescence, indicating intact mitochondrial membranes. The vehicle group (glucose 25 mM) shows increased green/yellow fluorescence, signifying mitochondrial depolarization. Treatment with saffron-based extracts—Repron^®^ saffron extract (sR, 40 µg/mL), hydroponic saffron extract (sH, 40 µg/mL), whole-saffron-flower extract (CsE, 150 µg/mL), and saffron tepal extract (TE, 150 µg/mL)—preserves mitochondrial membrane potential. Cell nuclei are counterstained with DAPI (blue). Scale bar = 25 µm. Panel (**B**) presents the ratio of MitoLight fluorescence (568/488 nm), normalized to CTRL, quantified using ImageJ. Data are expressed as mean ± SEM (n=4) and analyzed via one-way ANOVA (** *p* < 0.01). The vehicle confirms the damaging effect of hyperglycemia on mitochondria, whereas cells treated with saffron extracts exhibit partially restored membrane potential, supporting a protective role for these extracts under hyperglycemic conditions.

**Figure 10:**
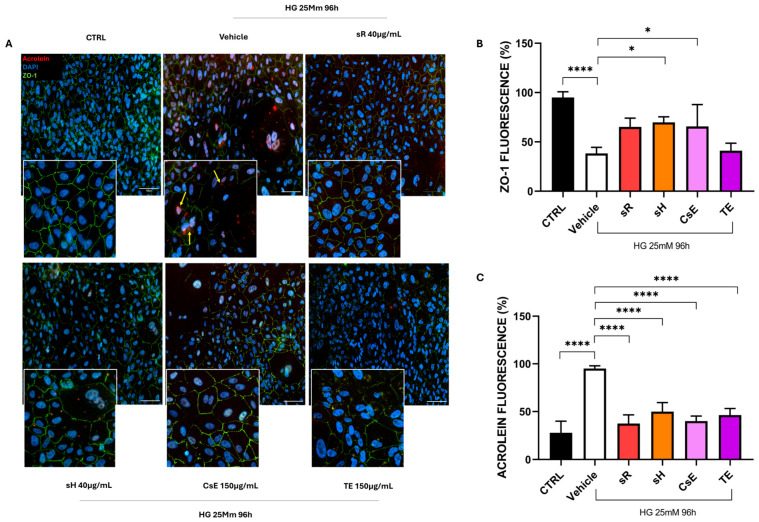
Saffron extracts minimize acrolein accumulation and support tight junction integrity under hyperglycemic stress. (**A**) presents the immunofluorescence of acrolein (red) and ZO-1 (green) in cells cultured with 25 mM glucose for 96 h. Acrolein is a highly reactive aldehyde generated during lipid peroxidation, recognized as a marker of oxidative damage. ZO-1 is a critical protein involved in tight junction assembly and barrier maintenance. Compared with the CTRL group (normoglycemic condition), the vehicle (glucose 25 mM) exhibits elevated acrolein signals (acrolein adducts are indicated by the yellow arrows) and disrupted ZO-1 localization. In contrast, cells treated with Repron^®^ saffron extract (sR, 40 µg/mL), hydroponic saffron extract (sH, 40 µg/mL), whole-saffron-flower extract (CsE, 150 µg/mL), or saffron tepal extract (TE, 150 µg/mL) display reduced acrolein staining and more continuous ZO-1 at cell borders. Nuclei are counterstained with DAPI (blue). Scale bar = 25 µm. (**B**) quantifies ZO-1 fluorescence intensity to gauge tight junction preservation, while (**C**) measures acrolein fluorescence to indicate oxidative stress levels. Data are shown as mean ± SEM (n = 4) and analyzed using one-way ANOVA (* *p* < 0.05; **** *p* < 0.0001).

**Figure 11:**
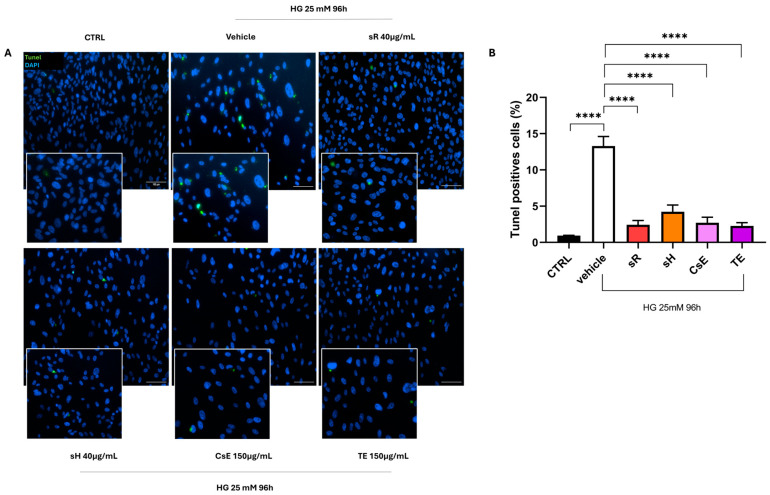
Saffron extracts reduce hyperglycemia-induced apoptosis: TUNEL assay. (**A**) displays TUNEL staining (green) in cells grown under high glucose (25 mM) conditions for 96 h, with nuclei counterstained by DAPI (blue). The CTRL (normoglycemic) group exhibits minimal apoptotic labeling, while the vehicle group (glucose 25 mM) shows a marked increase in TUNEL-positive cells, indicating increased apoptosis. Treatment with Repron^®^ saffron extract (sR, 40 µg/mL), hydroponic saffron extract (sH, 40 µg/mL), whole-saffron-flower extract (CsE, 150 µg/mL), or saffron tepal extract (TE, 150 µg/mL) mitigates this effect, resulting in fewer TUNEL-positive cells. Scale bar = 100 µm. (**B**) quantifies the percentage of TUNEL-positive cells (mean ± SEM, n = 4). Statistical analysis by one-way ANOVA confirms that saffron extract treatments significantly decrease apoptosis compared to vehicle (**** *p* < 0.0001).

Finally, Figure 9 shows the results obtained in the cellular model of hyperglycemia. The healthy control (CTRL) cells predominantly exhibit red labeling, indicating preserved mitochondrial membrane integrity. In contrast, damaged cells treated only with vehicle show predominantly green labeling, suggesting that hyperglycemia also triggers mitochondrial damage and dysfunction. The labeling pattern varies depending on the extract source in the panels displaying different treatments, but cells treated with sR, sH, CsE, and TE exhibit increased red labeling. These observations, summarized in Panel B, indicate that under hyperglycemia-induced stress, the extracts partially protect mitochondrial integrity and functionality. Figure 10A displays ARPE-19 cells stained in green for ZO-1 and in red for acrolein. Panels B and C show the quantification of ZO-1 and acrolein fluorescence. Acrolein, a toxic aldehyde produced by cells as a by-product of lipid metabolism, is used as a specific marker due to its relevance for its toxic effects and its role in cellular processes related to oxidative stress. Under hyperglycemia, acrolein plays a significant role due to its toxic effects associated with lipid peroxidation and cellular damage. CTRL cells show only sporadic acrolein staining, indicating a proper physiological state for the cells. In contrast, cells damaged by hyperglycemia and treated with vehicle alone show intense red staining, indicating a high rate of lipid peroxidation and, thus, oxidative stress. In the images of cells treated with the various types of extracts, acrolein staining is sporadic (as for the CTRL cells), indicating that all extracts used can protect the cells from this specific type of cellular stress. This result is confirmed by the quantification of acrolein fluorescence (Figure 10C), where the bar graph shows that all extracts significantly reduce acrolein production. The protective capacity of saffron extracts against hyperglycemia-induced damage is confirmed by the TUNEL analysis. Figure 11 shows that cells treated with any of the analyzed extracts are protected from the apoptotic process, reducing the percentage of TUNEL+ cells (Figure 11B).

## 3. Discussion

The data obtained in this study show that saffron Repron^®^ (ground and hydroponic) can preserve cells from various stress conditions. Moreover, it confirmed that extracts obtained from saffron by-products also retain a certain degree of protective efficacy against different types of cellular damage. To correlate the chemical composition of saffron extracts with the observed biological effects, the discussion is divided into two main perspectives based on the nature of the molecules found in the various extracts.

### 3.1. Flavonoids and Mitochondrial Protection

Saffron by-product extracts (CsE and TE) were found to be rich in glycosylated flavonoids, such as kaempferol, isorhamnetin, and quercetin. These compounds are well known for their antioxidant and anti-inflammatory properties, which explain their ability to preserve mitochondrial integrity under stress conditions (H_2_O_2_, LPS, and hyperglycemia) [33,34]. The greatest protective effect was observed for saffron Repron^®^ (sR), which exhibited the highest retention of red staining in mitochondrial viability assays, suggesting greater mitochondrial membrane stability, probably due to the high and characteristic crocin and crocetin content in this spice [6]. A comparable level of protection was also observed following treatment with extracts derived from secondary plant materials, particularly CsE, which is notably rich in kaempferol. In the oxidative stress model, CsE exhibited a statistically significant effect equivalent to that of Repron^®^. The observed protective effect is consistent with the activity of kaempferol in activating the Nrf2 signaling pathway, promoting antioxidant enzyme expression, and reducing inflammation, thus preserving mitochondrial function [35]. Our tests also show that acrolein (a marker of oxidative stress and lipid peroxidation) accumulation is significantly reduced in the presence of all extracts, indicating a common protective effect. This effect is particularly evident for extracts with high flavonoid and phenolic derivative content, which are known for their role in scavenging free radicals (e.g., superoxide and hydroxyls) [36,37] and modulating antioxidant pathways, such as SIRT1 activation. In this context, quercetin has been shown to reduce oxidative stress-induced apoptosis via this mechanism, further supporting the involvement of flavonoids in key cellular stress responses [38]. The reduced apoptosis in the TUNEL assays confirms the link between chemical composition and cellular protection, with the best results obtained from sR and sH extracts, followed by CsE and TE.

### 3.2. Flavonoids, Anthocyanins, and Integrity of Cellular Junctions

Tepal extracts (TEs) contain anthocyanins, such as delphinidin and petunidin, in glycosylated form. Anthocyanins protect the epithelial barrier by modulating the expression of junctional proteins, such as ZO-1 [39,40]. Moreover, flavonoids enhance tight junction integrity by promoting the expression and membrane localization of proteins, such as ZO-1, ZO-2, occludin, and claudins. These effects are probably mediated through the modulation of intracellular signaling pathways, including MAPKs and PKC, which play central roles in maintaining epithelial barrier function [41,42,43]. However, biological results show that TE—despite having some protective effects—does not reach statistically significant levels in preserving cellular junctions compared with other extracts (sR, sH, CsE). This suggests that glycosylated flavonoids (which are more abundant in sR and CsE) may have a more direct effect on the structural protection of cells. The data obtained confirm that saffron and extracts derived from its by-products exert significant protective effects against various types of cellular stress. Chemical and biological analyses suggest that this activity in by-product extracts can be mainly attributed to the presence of glycosylated flavonoids and anthocyanins through distinct mechanisms of action. In particular, flavonoid-rich extracts (CsEs) demonstrate greater efficacy in mitochondrial protection, reducing oxidative damage, and modulating antioxidant pathways, such as SIRT1 activation [38]. The maintenance of mitochondrial integrity and reduced acrolein accumulation support the role of these compounds in stabilizing the mitochondrial membrane and preventing apoptosis. However, anthocyanin-rich extracts (TEs) show a protective effect on cellular junctions, albeit less pronounced than that mediated by flavonoids. This suggests that, while anthocyanins contribute to epithelial barrier stability, glycosylated flavonoids may have a more significant impact on the structural protection of cells.

Overall, our results highlight the potential of saffron and its by-product extracts as nutraceutical resources to counteract oxidative and inflammatory damage and preserve cell function and morphology, with possible diverse applications in the context of neurodegenerative and metabolic diseases.

Although the extracts can potentially be used as a supplement to mitigate and reduce oxidative stress and inflammatory status, their mechanism of action remains unclear.

## 4. Materials and Methods

### 4.1. Chemicals and Reagents

UHPLC-grade methanol, water, and formic acid were purchased from Merck KGaA (Darmstadt, Germany). All analytical grade solvents were purchased from VWR (Milano, Italy).

### 4.2. Plant Material and Extract Preparation

Saffron flowers, deprived of their stigmas at the time of harvesting and considered agricultural waste, were provided by the Tuscany “Montegrappa Farm by Anastasia Vecchiarelli” (Grosseto, Italy). The fresh biomass (3.17 kg) was extracted via static maceration with 5 L of a mixture of EtOH/H_2_O (3:2 *v*/*v*) immediately after harvesting. The extracting solution was filtered, and the solvent was removed under vacuum to obtain a dry extract. In total, 1 g of the dry extract was partitioned with a mixture of *n*-BuOH/H_2_O (1:1 *v*/*v*) to remove sugars. The *n*-butanol phase was evaporated to obtain a dry residue (CsE).

Tepals (Navelli) were dried in an oven at 50 °C for 2 h and then placed in a flask and minced. Then, 100 mg of powder was weighed and placed in contact with extracting mixtures: H_2_O/EtOH (1:1, *v*/*v*). The extraction was carried out under magnetic stirring at room temperature for 1 h. The samples were placed in a centrifuge at 3000 rpm for 5 min. The supernatant was removed and filtered with a 0.20 µm nylon filter.

### 4.3. Chemical Characterization of Extracts

#### 4.3.1. UHPLC-DAD-HR-Orbitrap/ESI-MS^2^ Analyses

Chemical analyses of CsE and TE (2 mg/mL, methanolic solutions) were performed via Ultra-High-Performance Liquid Chromatography (UHPLC; Vanquish Flex Binary pump) coupled with a diode array detector (DAD) and a high resolution (HR) Q Exactive Plus Mass Spectrometry (MS), based on Orbitrap technology, equipped with an electrospray ionization (ESI) source (Thermo Fischer Scientific Inc., Bremen, Germany). Chromatographic analyses were performed using a Kinetex^®^ Biphenyl C-18 column (2.1 × 100 mm, 2.6 μm) equipped with a Security Guard^TM^ Ultra Cartridge (Phenomenex, Bologna, Italy) at a flow rate of 0.5 mL/min, using a splitting system of 1:1 attached to an MS detector (250 µL/min) and a DAD/UV detector (250 µL/min), respectively. The autosampler and the column oven were maintained at temperatures of 4 °C and 35 °C, respectively, and the injection volume was 5 μL. The elution was performed using a mixture of H_2_O/HCOOH 0.1% *v*/*v* (solvent A) and MeOH/HCOOH 0.1% (solvent B) according to a linear gradient of 5 to 80% (B) within 20 min. DAD data were registered in a 200–600 nm range using four preferential channels at 254, 280, 325, and 520 nm for phenols and anthocyanins. Nebulization voltage of 3500 V, capillary temperature of 300 °C, sheath gas (N_2_) of 20 arbitrary units, auxiliary gas (N_2_) of 3 arbitrary units, and HCD (higher-energy C-trap dissociation) of 18 eV were applied as ionization settings. To analyze the specialized metabolites in the plant extracts, a scan range of *m*/*z* 135–2000 was applied, recording MS both in full (70,000 resolution, 220 ms maximum injection time) and in a data-dependent MS/MS scan (17,500 resolution, 60 ms maximum injection time).

#### 4.3.2. HPLC-PDA Analysis of Anthocyanins

To characterize anthocyanins, we used a reversed-phase HPLC system equipped with a photodiode array UV–Vis detector and a C18 5 µm column (Kinetex EVO, Phenomenex, Bologna, Italy). The eluent phase comprised deionized and demineralized water acidified with 0.1% phosphoric acid (solvent A) and acetonitrile (solvent B). The gradient used was as follows: initial condition of 95% B and 5% A, reaching 100% B in 30 min. The chromatograms were acquired at 520 nm, with typical anthocyanin absorbance.

### 4.4. Cell Culture

ARPE-19 cells (ref. CRL-2302, ATCC Inc., Manassas, VA, USA) were grown in a 1:1 mixture of Dulbecco’s Modified Eagle Medium (DMEM) and Nutrient Mixture F-12 Ham, supplemented with 10% fetal bovine serum (FBS) and 1% penicillin–streptomycin. The cell cultures were maintained at 37 °C in a humidified atmosphere with 95% O_2_ and 5% CO_2_. The materials used for cell culture were obtained from Sigma-Aldrich (Merck, Darmstadt, Germany).

#### 4.4.1. Pretreatment Protocol

The cells were plated in a 96-well plate at a density of 1 × 10^4^ cells per well and incubated at 37 °C in an atmosphere containing 5% CO_2_. After 4 days, the cells were pretreated for 24 h with 40 µg/mL of saffron Repron^®^ (sR), hydroponic saffron (sH), and 150 µg/mL of tepal extracts (TEs) and waste extracts (CsEs). All treatments were diluted in culture medium and filtered using a syringe filter (Filtropur S, TES, pore size 0.2 µm).

#### 4.4.2. Retinal Degeneration Models

**Figure 12:**
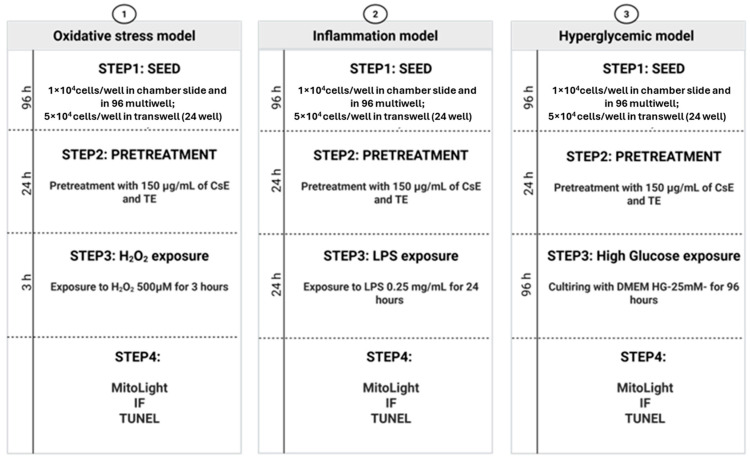
Experimental protocols. In all protocols, ARPE-19 cells were pretreated with the two different extracts for 24 h: the waste extract (CsE) and tepal extract (TE). The saffron Repron^®^ (sR) and hydroponic saffron (sH) were used as positive controls. In the oxidative stress model (protocol 1), after 24 h of pretreatment, cells were exposed to 500 µM H_2_O_2_ for 3 h, together with the extracts. In the inflammation model (protocol 2), after pretreatment, cells were exposed to 0.25 mg/mL of LPS for 24 h. In the hyperglycemic model (protocol 3) cells were pretreated with the extracts for 24 h, and then, the specific culture medium was replaced with DMEM HG (glucose 25 mM), supplemented with the extracts; DMEM HG was maintained for 96 h. At the end of each protocol, different analyses were performed.

We tested the extract’s protective efficacy in three different retinal degeneration models using three experimental protocols (Figure 12): (1) H_2_O_2_ protocol: After 24 h of pretreatment, 500 µM H_2_O_2_ (Sigma-Aldrich, Merck, Darmstadt, Germany**,** starting concentration 0.89 M) was added to the wells and maintained for 3 h at 37 °C in 5% CO_2_ before a biochemical assay. (2) LPS Protocol: Lipopolysaccharide (LPS) derived from *Escherichia coli* was obtained from Sigma-Aldrich. A stock solution of LPS (1 mg/mL) was prepared in physiological saline and subsequently diluted to a working concentration of 0.25 mg/mL in DMEM-F12 (Merck, Darmstadt, Germany; cod. D6421). Cells were pretreated with extracts for 24 h as described above and subsequently exposed to LPS for 24 h before carrying out biochemical assays. (3) HG protocol: Cells were pretreated with extracts for 24 h. To induce high glucose stress, cells were incubated for 96 h by replacing the specific culture medium with Dulbecco’s modified Eagle’s medium-high glucose (DMEM-HG**;** Merck, Darmstadt, Germany; cod. D5671) supplemented by 10% fetal bovine serum (FBS**;** Merck, Darmstadt, Germany; cod. F9665), 1% penicillin–streptomycin (Merck, Darmstadt, Germany; cod. P4333), and the extracts at the concentration described above.

### 4.5. TUNEL Assay

Cells were seeded into an 8-well chamber slide at a density of 1 × 10^4^ cells per well and treated following the three previously described protocols. The cells were then fixed with 4% paraformaldehyde for 25 min at 4 °C and permeabilized using 0.2% Triton^®^ X-100 for 5 min. The nuclear DNA fragmentation was evaluated with a TUNEL apoptosis detection kit (DeadEnd™ Fluorometric TUNEL system, Promega, Madison, WI, USA) according to the supplier’s instructions. Nuclei were stained with DAPI (Sigma-Aldrich, Merck, Darmstadt, Germany) at a 1:5000 dilution in phosphate-buffered saline (PBS). Finally, the cells were imaged using a Nikon Ni-E fluorescence microscope (Nikon Instruments Inc., Melville, NY, USA) equipped with a DS-Ri2 camera and analyzed via ImageJ (Bethesda, MD, USA).

### 4.6. Immunofluorescence

Cells were plated in an 8-well chamber slide at a density of 1 × 10^4^ cells per well and treated according to the previously outlined protocols. After treatment, the cells were fixed with 2% paraformaldehyde for 15 min at room temperature, permeabilized with a solution containing 2.5% bovine serum albumin (BSA) and 0.3% Triton X-100 for 10 min, and then blocked with 2.5% BSA for 1 h. The cells were incubated overnight at 4 °C with primary antibodies against ZO-1 (Invitrogen, cod. 61–7300, Thermo Fisher Scientific, Waltham, MA, USA, 1:500) and acrolein (Abcam, cod. ab48501, Cambridge, UK, 1:1000). The next day, the cells were exposed to the appropriate fluorescent secondary antibodies for 2 h at room temperature. Finally, the cells were counterstained with DAPI, washed three times with PBS, imaged, and analyzed as previously described.

### 4.7. Detection of Mitochondrial Membrane Potential

Cells were seeded in an 8-well chamber slide at a density of 1 × 10^4^ cells per well and treated as previously described. To assess the protective effect of CsE and TE, cells were stained using the MitoLight Mitochondrial Apoptosis Detection Kit (Millipore, Burlington, MA, USA) for 15 min at 37 °C. After staining, cells were fixed with 2% paraformaldehyde (PFA) for 15 min, followed by three washes with PBS. Cell nuclei were then stained with DAPI. The MitoLight dye behaves differently in healthy and apoptotic cells: in healthy cells, it accumulates in the mitochondria and emits bright red fluorescence. In apoptotic cells, where the mitochondrial membrane potential is altered, the dye remains in its monomeric form in the cytoplasm and shows green fluorescence, allowing for easy differentiation between apoptotic and non-apoptotic cells. Finally, cells were imaged and analyzed as previously described.

### 4.8. Image Acquisition and Quantitative Analysis

The same method of image acquisition and fluorescence analysis was applied across all microscopy-based assays (TUNEL, immunofluorescence, and mitochondrial membrane potential detection). For each well, between 6 and 8 different fields were acquired. The fields were selected by moving systematically from left to right and then downward across the well. This predefined scanning path was used in all replicates to minimize operator bias and to avoid the possibility of analyzing the same field multiple times. This method was designed to balance consistency with a degree of randomness in the field selection.

Fluorescence analysis was performed using ImageJ. Each image was divided into four equal rectangular regions using the rectangle selection tool, and measurements were performed independently for each sub-region. The resulting values were then averaged to obtain a representative signal for the entire image.

The measurement parameters were set to include area, mean gray value, and integrated density. The mean gray value was used to determine the average fluorescence intensity per pixel. Additionally, for each image, a background area free of a specific fluorescence signal was selected and measured. The background value was subtracted from all fluorescence intensity measurements to correct for the non-specific signal.

### 4.9. Statistical Analyses

Each experiment was repeated at least three times for each protocol. The data obtained were analyzed using GraphPad Prism version 8.0 for Windows (GraphPad Software, San Diego, CA, USA). The results for each experimental group were compared using one-way ANOVA. Prior to analysis, the Brown–Forsythe test was performed to assess the homogeneity of variances. The results obtained for each experimental group were normalized to those obtained for the healthy control group (no treatment or exposure to H_2_O_2_, LPS, or high glucose concentrations). All groups are presented as mean ± SEM. *p*-values less than 0.05 were considered statistically significant.

## 5. Conclusions

Extracts of saffron and by-products from its production exhibit different biological effects depending on their chemical composition. Glycosylated flavonoids, which are abundant in TEs and CsEs, appear to be responsible for mitochondrial protection and the maintenance of cell junction integrity. Anthocyanins (TEs) also contribute to epithelial barrier support, though to a lesser extent. All extracts reduced acrolein accumulation and apoptosis, indicating shared antioxidant and cytoprotective properties.

These findings suggest that saffron extracts, including those derived from processing waste, and especially those rich in glycosylated flavonoids, could be beneficial for retinal protection under the conditions of oxidative stress, inflammation, and hyperglycemia. Additionally, repurposing by-products may help to reduce production costs. However, further in vivo studies are needed to determine proper dosing and administration, although the historically favorable safety profile of saffron supports its potential clinical use.

## Figures and Tables

**Table 1:** Chromatographic data (retention time, *t*_R_) and HR-ESI-MS^2^ data of the annotated compounds in saffron extracts. The molecular formula and the calculated mass errors are also reported.

Peak	Compound	*t*_R_ (min)	[M − H]^−^ (*m*/*z*)	ESI-MS/MS (*m*/*z*)	Molecular Formula	Error (ppm)	Extract
***Organic acid***							
**1**	Citric acid	0.53	191.0192	173.01, 129.02, 111.01	C_6_H_8_O_7_	−2.62	CsE, TE
***Flavonoid glycosides***							
**2**	Kaempferol tri-*O*-glucoside	4.66	771.1974	609.14, 446.09, 285.04, 284.03	C_33_H_40_O_21_	−1.95	TE
**3**	Quercetin *O*-sophoroside	7.91	625.1414	545.05, 463.09, 301.04, 300.03	C_27_H_30_O_17_	0.640	CsE, TE
**4**	Kaempferol *O*-sophoroside	8.86	609.1466	429.08, 309.04, 285.04, 284.03	C_27_H_30_O_16_	0.821	CsE, TE
**5**	Quercetin *O*-glucoside	9.27	463.0883	301.04, 300.03, 271.02, 255.03	C_21_H_20_O_12_	0.220	CsE, TE
**6**	Kaempferol *O*-rutinoside	9.46	593.1516	429.08, 371.78, 285.04, 284.03	C_27_H_30_O_15_	0.674	CsE, TE
**7**	Isorhamnetin *O*-rutinoside	9.69	623.1621	594.16, 315.05, 314.04, 299.02	C_28_H_32_O_16_	0.481	CsE, TE
**8**	Kaempferol *O*-glucoside	10.38	447.0935	285.04, 284.03, 255.03, 227.03	C_21_H_20_O_11_	0.470	CsE, TE
**9**	Isorhamnetin *O*-glucoside	10.80	477.1038	357.06, 315.05, 314.04, 299.02	C_22_H_22_O_12_	−0.210	CsE, TE
**10**	Quercetin	11.93	301.0355	273.04, 193.01, 151.00, 93.03	C_15_H_10_O_7_	0.332	CsE, TE
**11**	Kaempferol	13.33	285.0405	151.00, 117.03	C_15_H_10_O_6_	0	CsE, TE
**12**	Isorhamnetin	14.16	315.0512	301.03, 279.86, 261.89, 219.85	C_16_H_12_O_7_	0.635	CsE, TE
***Fatty acids***							
**13**	Trihydroxyoctadecadienoic acid	14.25	327.2176	309.21, 291.20, 229.14, 211.13	C_18_H_32_O_5_	−0.31	CsE, TE
**14**	Trihydroxyoctadecenoic acid I	14.88	329.2332	311.22, 293.21, 249.22, 229.14	C_18_H_34_O_5_	0	CsE, TE
**15**	Trihydroxyoctadecenoic acid II	15.08	329.2332	311.22, 293.21, 249.22, 229.14	C_18_H_34_O_5_	0	CsE

CsE = saffron waste extract; TE = saffron tepal extract.

**Table 2:** Chromatographic data (retention time, *t*_R_) and HR-ESI-MS^2^ data of the annotated compounds in tepal saffron extracts. The molecular formula and the calculated mass errors are also reported.

Peak	Compound	*t*_R_ (min)	[M]^+^ (*m*/*z*)	ESI-MS/MS (*m*/*z*)	Molecular Formula	Error (ppm)	Extract
**16**	Delphinidin 3,5-di-*O*-glucoside	10.52	627.1558	465.10, 303.05	C_27_H_31_O_17_^+^	−0.637	TE
**17**	Petunidin 3,5-di-*O*-glucoside	11.13	641.1704	479.12, 317.06	C_28_H_33_O_17_^+^	−1.25	TE
**18**	Delphinidin 3-*O*-glucoside	11.46	465.1030	303.05, 287.06	C_21_H_21_O_12_^+^	0.430	TE
**19**	Petunidin 3-*O*-glucoside	12.06	479.1184	317.06	C_22_H_23_O_12_^+^	0	TE

## Data Availability

The experimental data that support the figures and other findings within this study are hosted at the Department of Pharmacy, University of Pisa, and can be accessed by contacting the corresponding author.
